# Supplementary material for: Continuous resin refilling and hydrogen bond synergistically assisted 3D structural color printing
Source: Nat Commun. 2022 Nov 19;13:7095. doi: 10.1038/s41467-022-34866-6 (PMC9675848; doi:10.1038/s41467-022-34866-6)
Supplement: Supplementary file 1 — Supplementary Information [file 41467_2022_34866_MOESM1_ESM.pdf]

SUPPLEMENTARY INFORMATION FOR

Continuous Resin Refilling and Hydrogen Bond Synergistically Assisted 3D Structural Color Printing

Yu Zhang,[1,2] Lidian Zhang,[1,2] Chengqi Zhang,[3] Jingxia Wang,[2,4] Junchao Liu,[4] Changqing Ye,[5] Zhichao Dong,[2,4] Lei Wu,[1,2*] Yanlin Song[1,2*]

[1]Key Laboratory of Green Printing, Beijing National Laboratory for Molecular Sciences (BNLMS), Institute of Chemistry, Chinese Academy of Sciences, Beijing, 100190, P. R. China.

[2]University of Chinese Academy of Sciences, Beijing, 100049, P. R. China.

[3]Beihang University, Beijing, 100191, P. R. China.

[4]Key Laboratory of Bio-inspired Materials and Interfacial Science, Technical Institute of Physics and Chemistry, Chinese Academy of Sciences, Beijing, 100190, P. R. China.

[5]School of Chemistry, Biology and Materials Engineering, Suzhou University of Science and Technology, Suzhou, 215009, P. R. China.

*Corresponding authors. E-mail: wulei1989@iccac.ac.cn; ylsong@iccas.ac.cn

Supplementary Figures

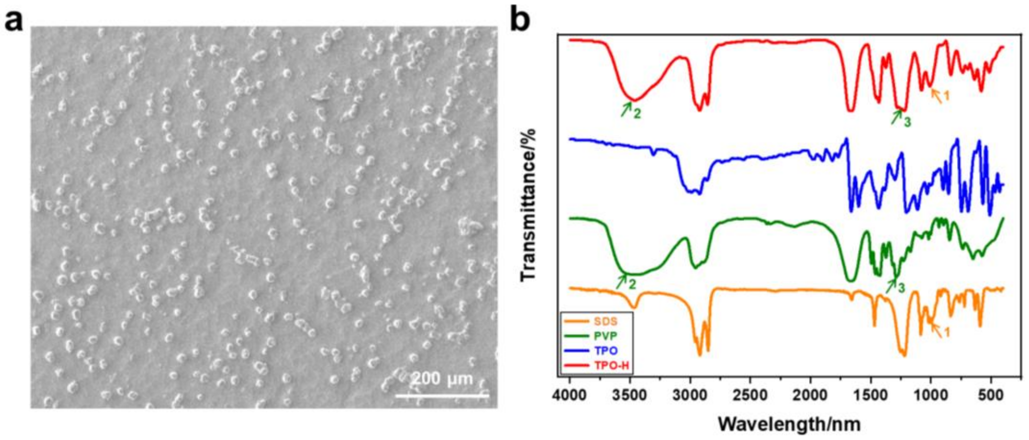

Supplementary Figure 1. Characterization of the hydrosoluble diphenyl(2,4,6-trimethylbenzoyl)phosphie oxide (TPO-H) photoinitiator. (**a**) SEM image of the synthesized TPO-H. (**b**) Fourier transform infrared spectrum of sodium dodecyl sulfate (SDS), polyvinylpyrrolidone (PVP), diphenyl(2,4,6-trimethylbenzoyl)phosphie oxide (TPO) and TPO-H. The numbers 1-3 represent the characteristic peaks of different components. In detail, 1 represents the stretching vibration peak of sulfate in SDS. 2 and 3 represent the stretching vibration peak of hydroxy and C-N in PVP, respectively. Compared to the commercially available TPO, the appearance of the characteristic peaks on the TPO-H indicates the successful encapsulation of TPO inside the PVP and SDS.

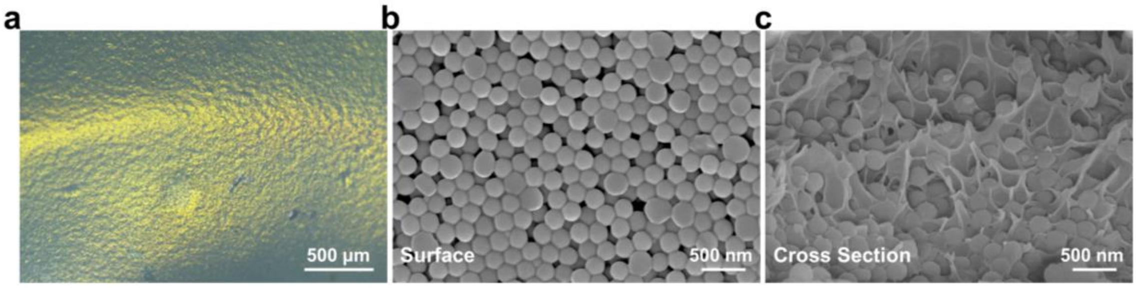

Supplementary Figure 2. Characterization of the directly cured structure from the UV-curable structural color ink containing 214 nm PS latex particles. (**a**) Optical image of the directly cured structure. (**b**) Surface SEM image of the directly cured structure. (**c**) Cross-sectional SEM image of the directly cured structure. The existence of structural color and the uniform distribution of PS latex particles inside the polymer skeleton can be ascribed to the formation of hydrogen bonds between the surface -COOH of PS latex particles and the -$CONH_2$ of monomer AM.

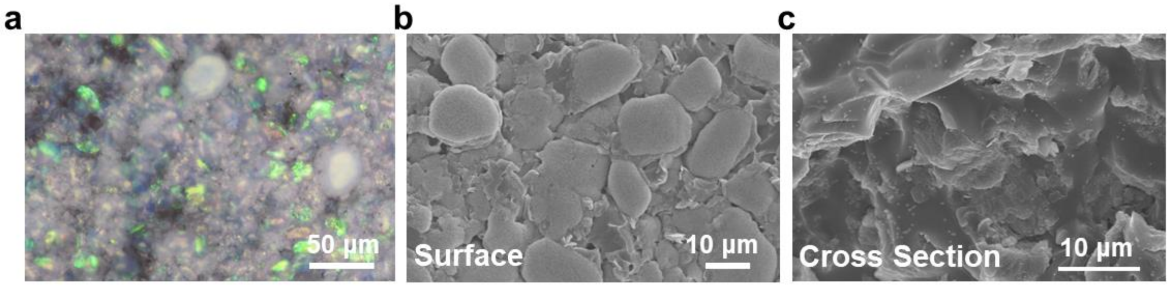

Supplementary Figure 3. Characterization of the slab structure fabricated with UV-curable structural color ink containing 214 nm PS latex particles and PEGDA as monomer through the continuous DLP 3D printing method. (**a**) Optical image of the 3D printed slab structure with PEGDA as monomer. (**b**) Surface SEM image of the 3D printed slab structure with PEGDA as monomer. (**c**) Cross-sectional SEM image of the 3D printed slab structure with PEGDA as monomer. Without the group that can form hydrogen bonds, phase separation occurs, which results in the PS micro-aggregates randomly distributing inside the polymer skeleton.

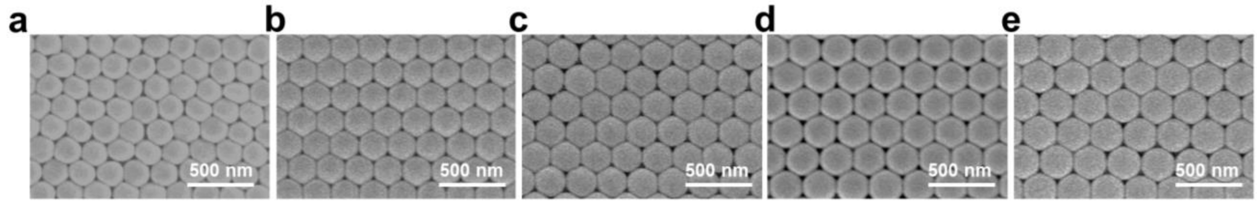

Supplementary Figure 4. SEM images of the assembly of pure PS latex particles with diameters of 192 nm (**a**), 214 nm (**b**), 230 nm (**c**), 245 nm (**d**) and 265 nm (**e**).

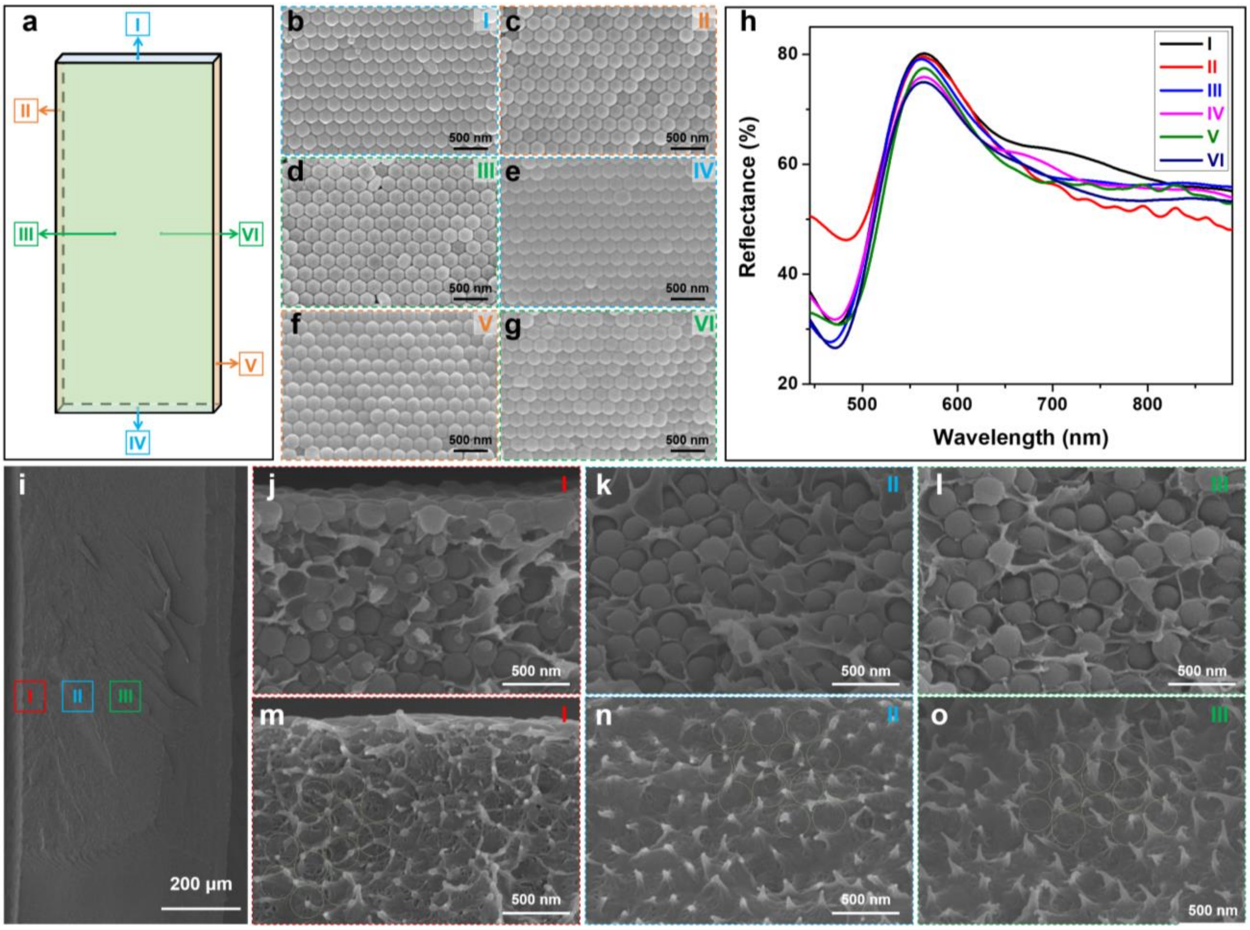

Supplementary Figure 5. The surface and internal assembly of latex particles after complete evaporation. (**a**) Scheme of the SEM characterization positions. I-VI represent the six different surfaces of the 3D printed slab structure. (**b-g**) SEM images of the I-VI surfaces in (**a**). (**h**) Reflectance spectra of the I-VI surfaces in (**a**). (**i**) Cross-sectional SEM image of the printed slab structure. I-III represent the different SEM characterization positions from the surface to the interior. (**j-l**) Cross-sectional SEM images of the different positions in (**i**). (**m-o**) Cross-sectional SEM images of the different positions in (**i**) after selectively removing the PS latex particles.

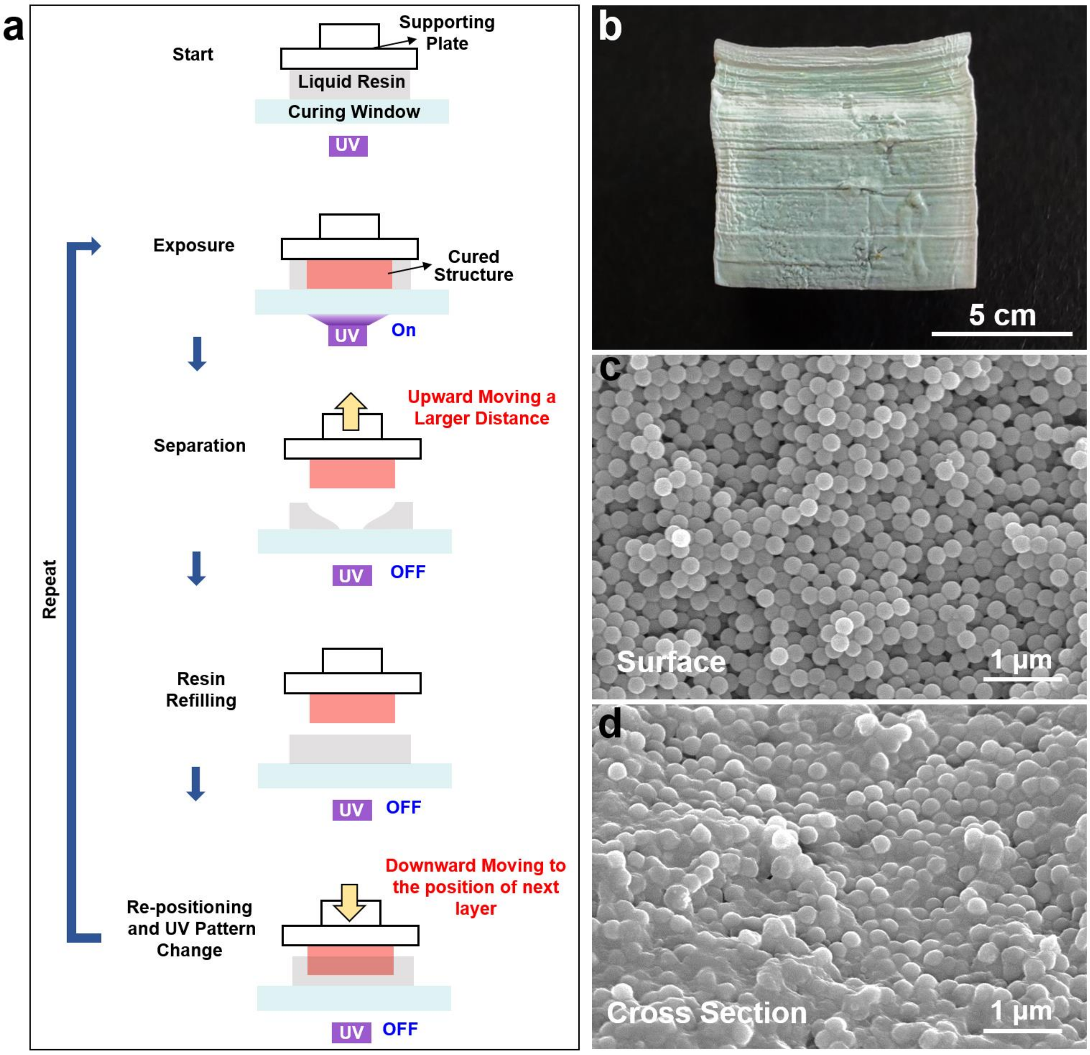

Supplementary Figure 6. Characterizations of the slab structure prepared with UV-curable structural color ink containing 214 nm PS latex particles through discontinuous printing process. (**a**) Scheme of the discontinuous DLP 3D printing process. As the resin refilling process is conducted separately and successively after upward lifting the supporting plate, no suction force occurs. (**b**) Optical image of the slab structure fabricated from the discontinuous printing process. The printed slab structure displays green-yellow structural color due to the hydrogen bonds formed between the UV-curable matrix AM and PS latex particles. (**c**) Surface SEM image of the slab structure fabricated from the discontinuous printing process. (**d**) Cross-sectional SEM image of the slab structure fabricated from

the discontinuous printing process. Without the ink filling induced by the continuous 3D printing, PS latex particles assemble randomly inside the polymer skeleton.

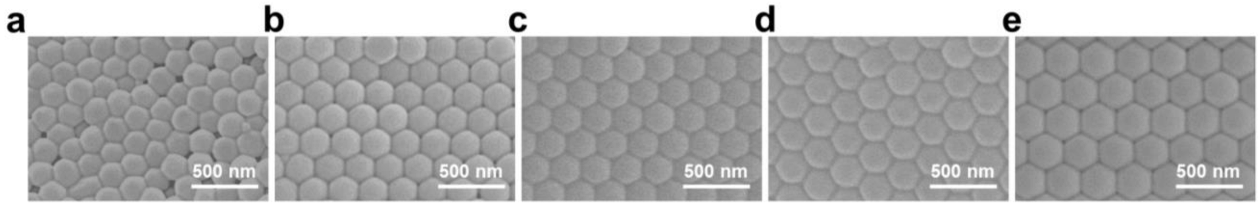

Supplementary Figure 7. Surface SEM images of the 3D PCs structures printed from the UV-curable structural color ink with PS latex particle diameters of 192 nm (**a**), 214 nm (**b**), 230 nm (**c**), 245 nm (**d**) and 265 nm (**e**).

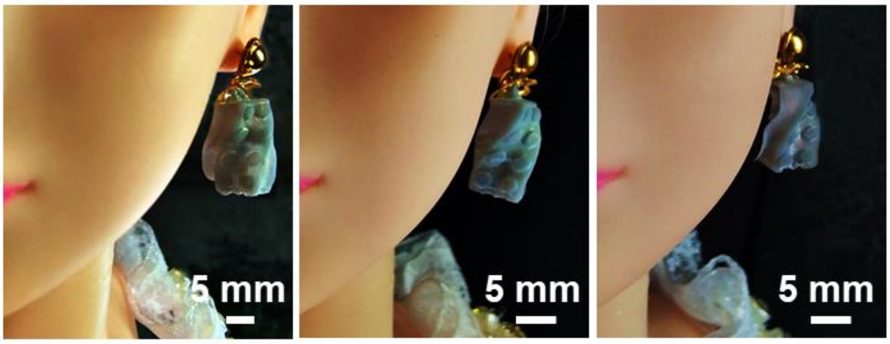

Supplementary Figure 8. Optical images of a Barbie doll wearing earring structure observed from different directions.

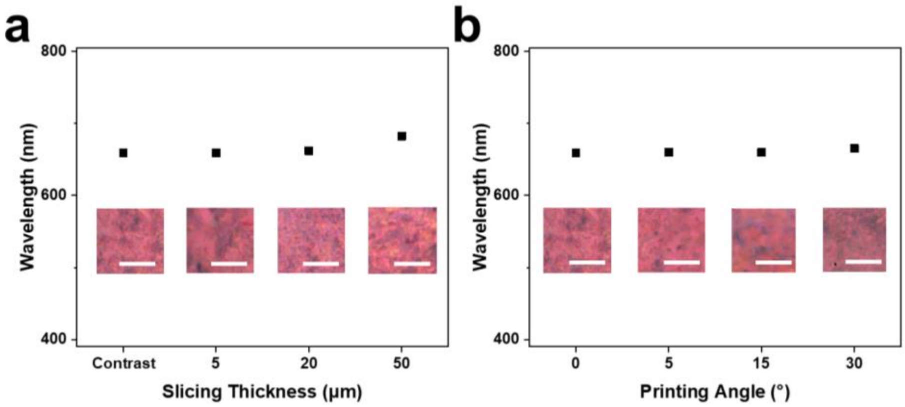

Supplementary Figure 9. The influence of printing parameters on the stopband wavelength. (**a**) The influence of slicing thickness on the stopband wavelength. Insets are optical images of the corresponding samples. Scale bars, 500 μm. (**b**) The influence of printing angle on the stopband wavelength. Insets are optical images of the corresponding samples. Scale bars, 500 μm. The influence of the slicing thickness and printing angle on the stopband wavelength can be ignored within the parameters used in the experiment.

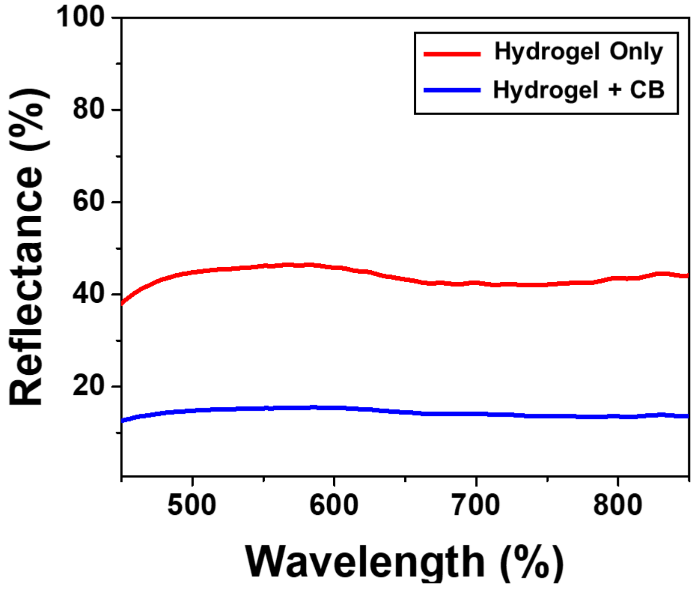

Supplementary Figure 10. Reflectance spectra of the 3D structures printed from the UV-curable system with additive carbon black (CB) and the pure UV-curable system. Neither of the above two structures has a characteristic reflection peak.

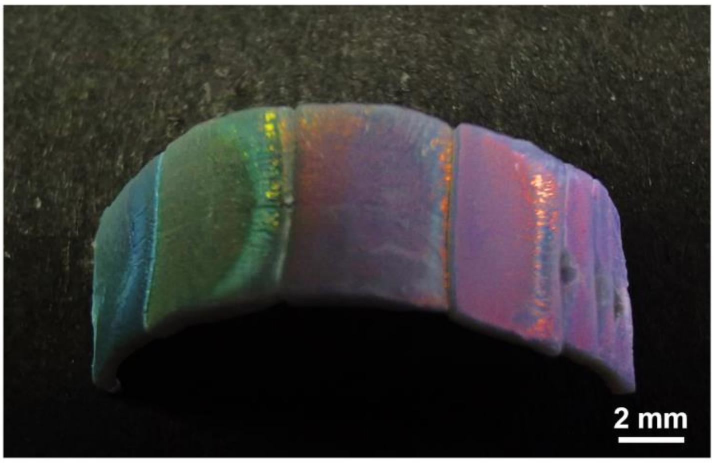

Supplementary Figure 11. Optical image of 3D printed structure with different structural colors. From left to right, the PS latex particle diameters used are 192 nm, 214 nm, 230 nm, 245 nm, and 265 nm, respectively.

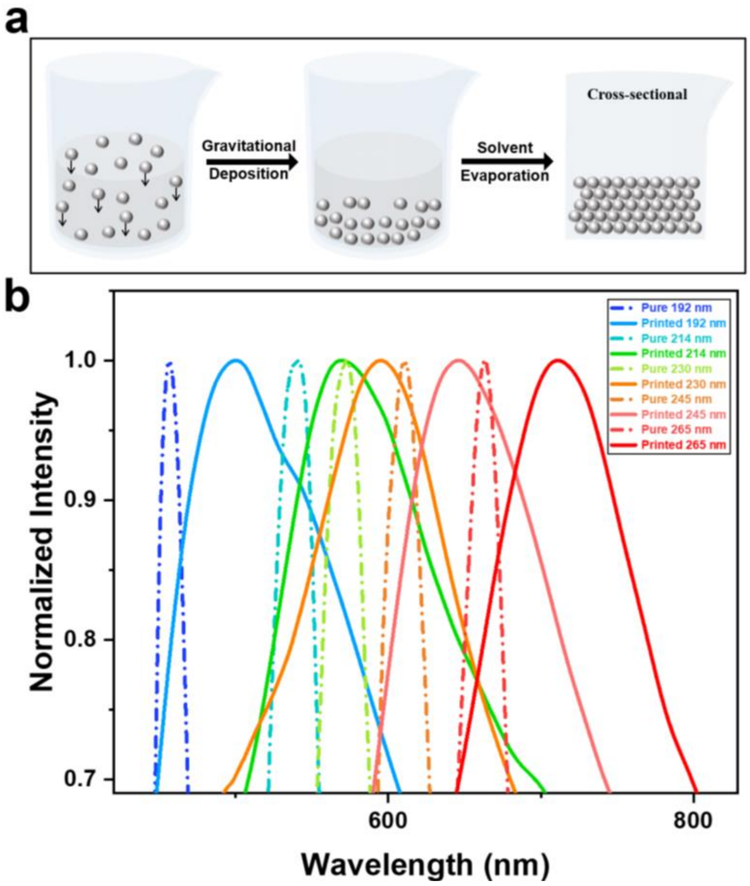

Supplementary Figure 12. Scheme of the gravitational deposition method (**a**) and the reflectance spectra of the pure PS latex particles assembly and 3D printed PCs structures (**b**). The PS latex particle diameters used are 192 nm, 214 nm, 230 nm, 245 nm and 265 nm. Dashed and solid lines represent the reflectance spectra of the pure PS latex particles assembled by the gravitational deposition method [1-4] at 20 °C and 20% relative humidity for ~ 8 h and the 3D printed PCs structure, respectively. Due to the existence of the polymer skeleton, the full width at half maximum becomes wider and the position of the stopband is correspondingly red-shifted. Comparing with the pure PS latex particles assembled by gravitational deposition method, the broadening of reflectance peak of the 3D printed structure can be ascribed to the high printing speed and assembly speed. In addition, the introduction of hydrogel interspersing the gaps among the PS latex particles may also lead to the broadening of the

reflectance peak than the pure latex particles assembly [5-9], which is a common phenomenon for the composite nanoparticle-hydrogel structures.

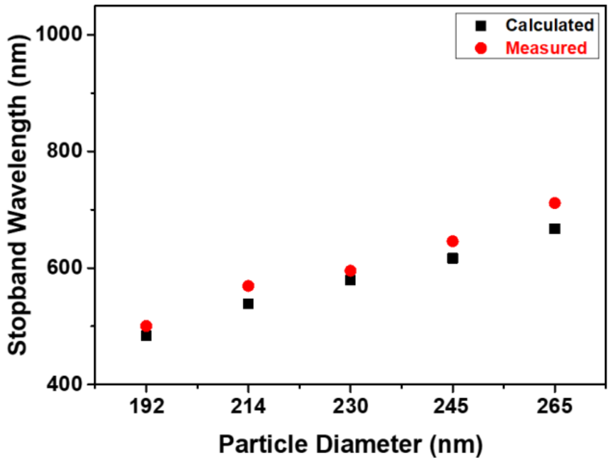

Supplementary Figure 13. The calculated and measured stopband wavelengths of the structures continuously 3D printed with different PS particle diameters.

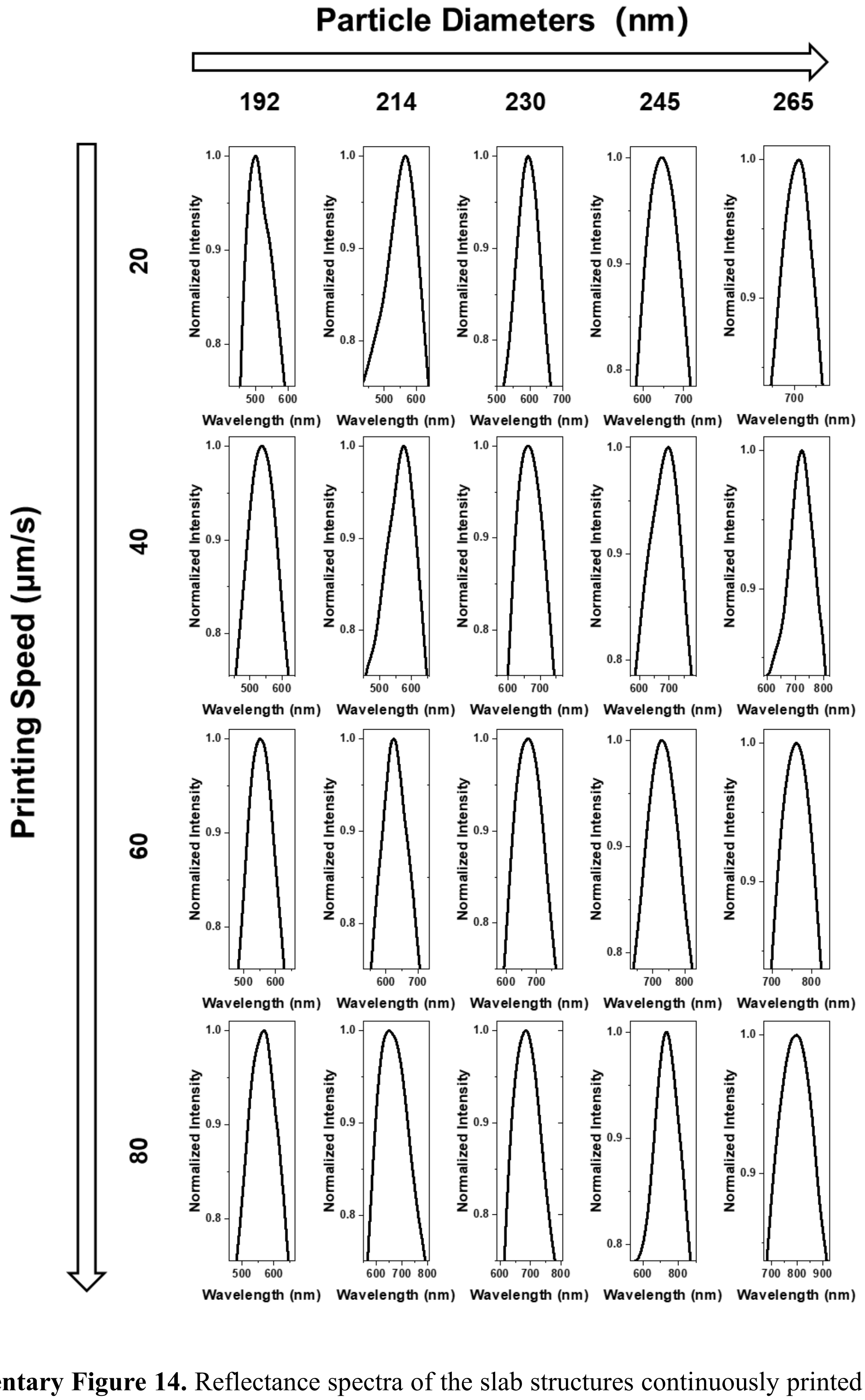

Supplementary Figure 14. Reflectance spectra of the slab structures continuously printed from UV-curable structural color ink with different PS latex particle diameters and printing speeds.

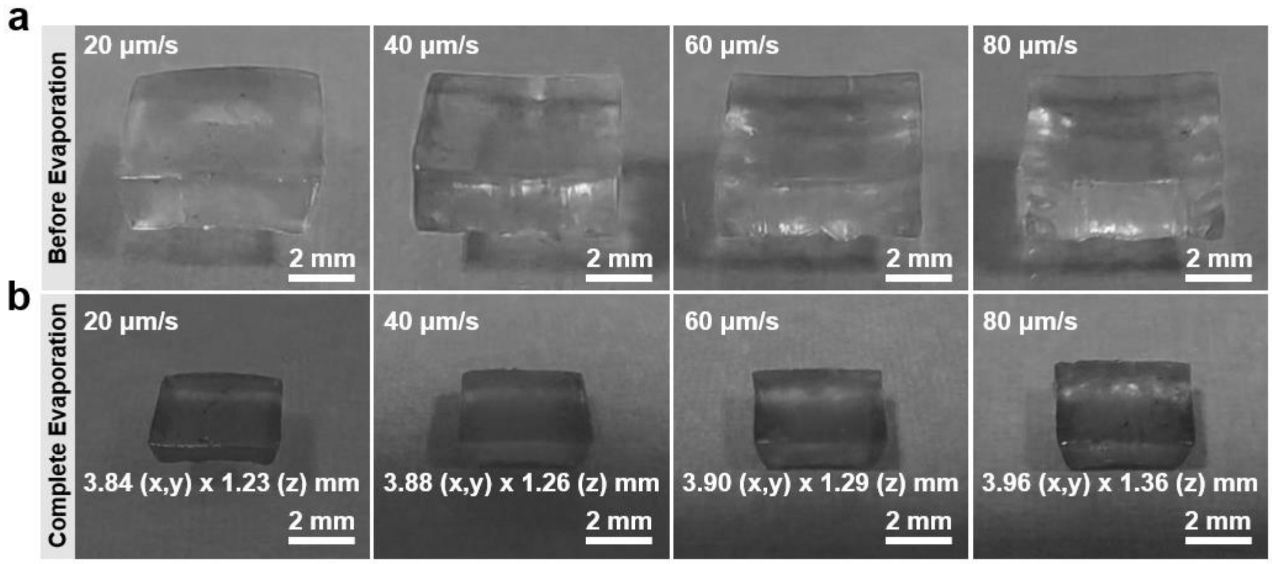

Supplementary Figure 15. Volumes of the pure AM hydrogels prepared under different printing speeds before (a) and after (b) complete evaporation. As the different printing speeds require different UV intensities and exposure times of one layer, which leads to different printing times for the same 3D structure. For example, to print a pure AM hydrogel structure with design of 6.0 mm × 6.0 mm × 2.0 mm (z-axis height), the speed of 20 μm/s needs 100.0 s under UV intensity of 5.7 mW/cm^2, the speed of 40 μm/s needs 50.0 s under UV intensity of 6.7 mW/cm^2, the speed of 60 μm/s needs 33.4 s under UV intensity of 7.2 mW/cm^2, while the speed of 80 μm/s requires 25.0 s under UV intensity of 7.6 mW/cm^2. For the different UV intensities and different printing times, the volumes before water evaporation are the same and correspond to designed volumes (**a**), while the dry volume after water evaporation for the sample cured fast under higher UV intensity is larger than that of the lower UV intensity (**b**). The results indicate that the higher printing speed under higher UV intensity will lead to larger dry volume of polymer skeleton and a smaller ratio of PS latex particles solidified inside the skeleton, resulting in a larger latex particle spacing and the red-shift of the stopband position.

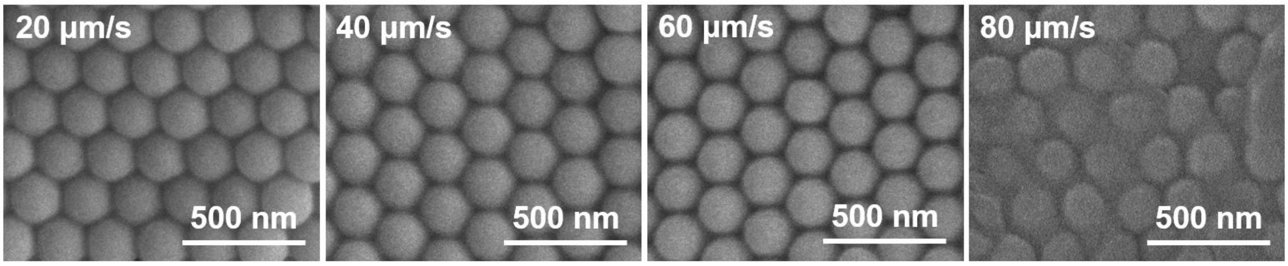

Supplementary Figure 16. Surface SEM images of the prepared PCs structure under different printing speeds.

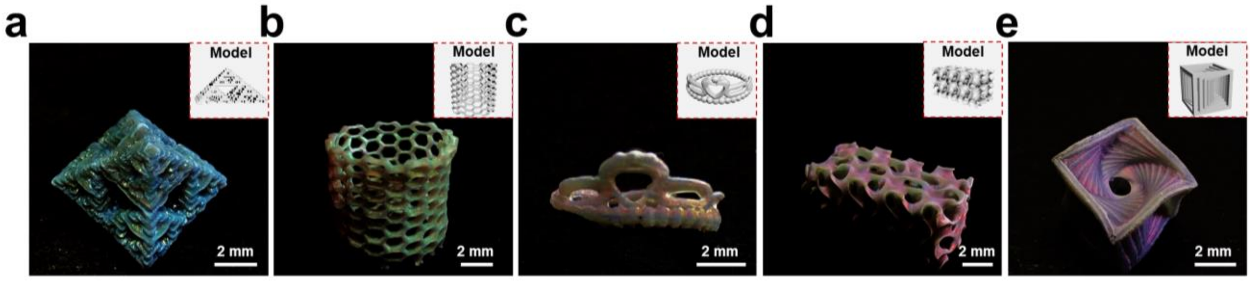

Supplementary Figure 17. 3D printing complex structures with desired structural colors. (**a**) Optical image of pyramid-like structure printed from 192 nm PS latex particle diameter. (**b**) Optical image of cylinder grid structure printed from 214 nm PS latex particle diameter. (**c**) Optical image of ring structure printed from 230 nm PS latex particle diameter. (**d**) Optical image of gyroid structure printed from 245 nm PS latex particle diameter. (**e**) Optical image of box with twisted internal structure printed from 265 nm PS latex particle diameter. Inset of each image is the 3D model of corresponding structure.

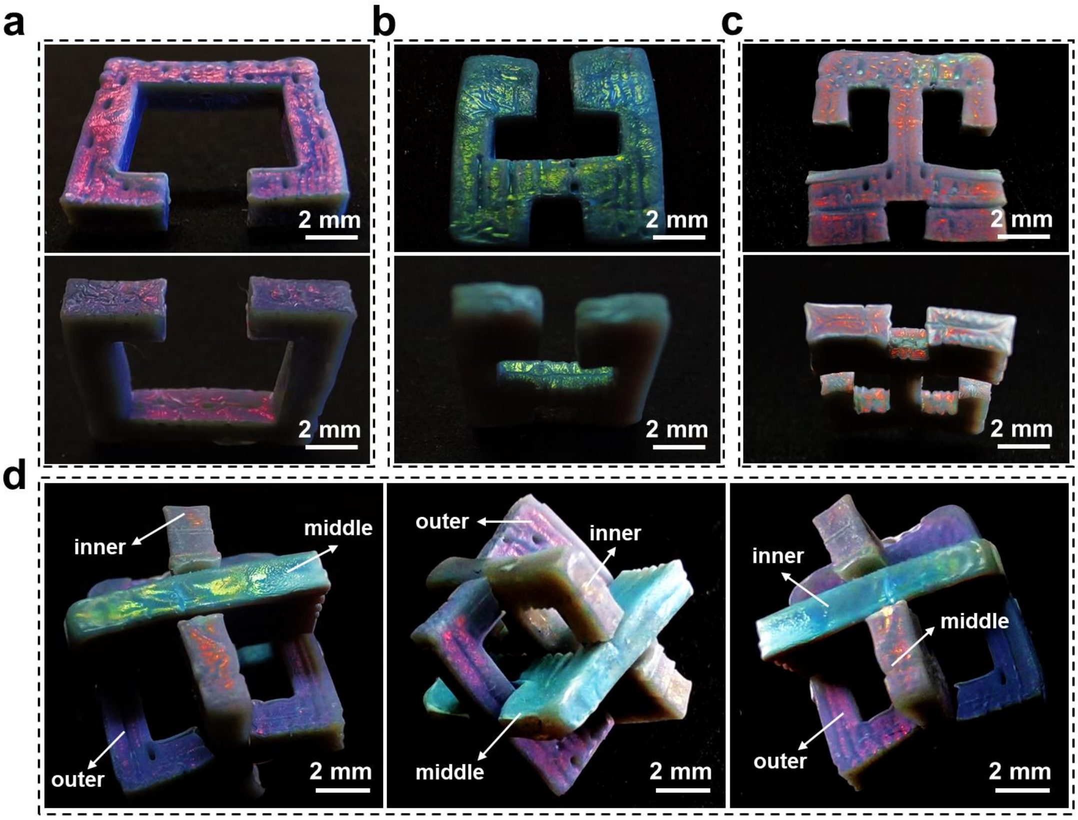

Supplementary Figure 18. Complex multi-structural colors 3D structure acquired through the assembly of different parts with single structural color. (**a**) Optical image of the outer part printed from 265 nm PS latex particle. (**b**) Optical image of the middle part printed from 192 nm PS latex particle. (**c**) Optical image of the inner part printed from 230 nm PS latex particle diameter. (**d**) Optical images of the assembled composite structure from the separated parts.

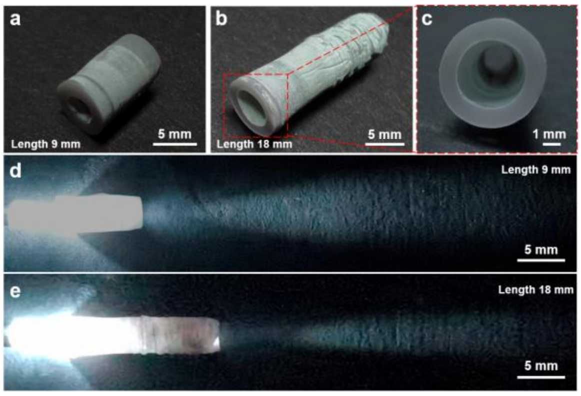

Supplementary Figure 19. Optical transportation property of the discontinuous DLP 3D printed PCs structures. (**a**, **b**) Optical images of the discontinuously printed hollow cylinder tube structures with lengths of 9 mm (**a**) and 18 mm (**b**), respectively. (**c**) Enlarged optical image of the through-hole in the red dashed box in (**b**). (**d**, **e**) Optical images of the discontinuously printed hollow cylinder-shaped tubes with lengths of 9 mm (**d**) and 18 mm (**e**), respectively.

Supplementary Table 1. Comparison of researches with this work concerning the preparation of 3D photonic crystal structure including preparation method, structural color generation mechanism and related-properties.

References		Facile full-color printing with a single transparent ink, Sci. Adv., 2021 [10]	Tunable structural color of bottlebrush block copolymers through direct-write 3D printing from solution, Sci. Adv., 2020 [11]	Structural color for additive manufacturing: 3D-printed photonic crystals from block copolymers, ACS Nano, 2017 [12]	Structural color three-dimensional printing by shrinking photonic crystals, Nat. Commun., 2019 [13]	3D-printable colloidal photonic crystals, Mater. Today, 2022 [14]	Our Work
Fabrication Process	3D Printing Method	Inkjet printing	Direct ink writing	Fused deposition modeling	Two-photon polymerization lithography	Discontinuous digital light processing printing	Continuous digital light processing printing
	Printing Process	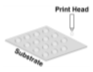	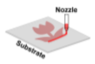	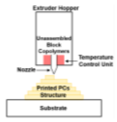	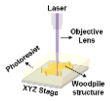	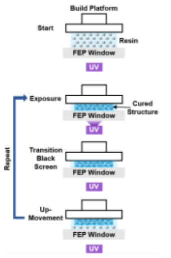	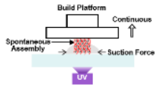
	Structural Color Generation Mechanism	Assembly during printing	Assembly before printing	Assembly during printing	Coloration after heat-induced shrinking	Assembly before printing	Assembly during printing
	Driving Force for Assembly	Surface tension	Capillary flow during drying	Thermally induced during filament extrusion	/	Electrostatic repulsive force between the highly charged elastic nanoparticles	Suction force induced by the continuous curing manner
	Structural Color Regulation Factors	Ink volume and substrate wettability	Printing speed and substrate temperature	Molecular weight of block copolymer	Lattice constants and laser power	Concentration of highly charged elastic nanoparticles, temperature and curing time	Particle diameter and printing speed
Structural Color Ink	3D Printable Ink	Transparent UV-curable polymer ink	Bottlebrush block copolymer solution	Dendritic block copolymers	Commercial acrylate-based photoresist	Acrylamide-based UV-curable ink with embedded HENPs	Acrylamide-based UV-curable ink with dispersed carboxyl-rich PS latex particles
	Structural Color Generation Principle	Total internal reflection from micro-dome	Reflection	Reflection	Bragg scattering	Bragg scattering	Bragg scattering
Structure Details and Properties	Resulting Structure	Plane shape 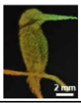	Plane shape 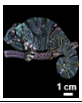	3D geometry 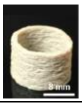	3D geometry 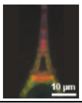	3D geometry 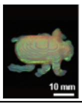	3D geometry 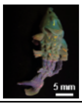
	Step Eliminating Effect	/	/	Lateral stripes and Rough surface	/	Lateral layered structure;	No steps; Smooth Surface

	**Printing Fidelity**	High fidelity	/	/	/	Limited fidelity due to the step structures and loss of structure continuity	High fidelity
	**State of the Final Structure**	Rigid	Rigid	Rigid	Rigid	Flexible and swollen equilibrium in water	Rigid
	**Volumetric color property**	/	/	/	√	/	√
	**Mechanical Property**	/	/	/	/	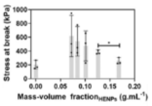<br>Enhanced mechanical strength	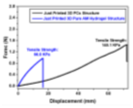<br>Enhanced mechanical strength

Supplementary Table 2. Optical losses of optical light-guide structures with different lengths.

**Length (mm)**	9	18	20
**Optical Loss (dB)**	5.37 ± 0.30	7.08 ± 0.17	7.65 ± 0.47

Supplementary References

1. Jiang, P., Bertone, J. F., Hwang, K. S. & Colvin, V. L. Single-crystal colloidal multilayers of controlled thickness. *Chem. Mater.* **11**, 2132-2140 (1999).
2. Huang, Y. et al. Colloidal photonic crystals with narrow stopbands assembled from low-adhesive superhydrophobic substrates. *J. Am. Chem. Soc.* **134**, 17053-17058 (2012).
3. Wang, J., Wen, Y., Feng, X., Song, Y. & Jiang, L. Control over the Wettability of Colloidal Crystal Films by Assembly Temperature. *Macromol. Rapid Commun.* **27**, 188-192 (2006).
4. von Freymann, G., Kitaev, V., Lotsch, B. V. & Ozin, G. A. Bottom-up assembly of photonic crystals. *Chem. Soc. Rev.* **42**, 2528-2554 (2013).
5. Takeoka, Y. et al. Production of colored pigments with amorphous arrays of black and white colloidal particles. *Angew. Chem. Int. Ed.* **52**, 7261-7265 (2013).
6. Fu, F., Shang, L., Chen, Z., Yu. Y. & Zhao, Y. Bioinspired living structural color hydrogels. *Sci. Robot.* **3**, eaar8580 (2018).
7. Kim, H. et al. Structural colour printing using a magnetically tunable and lithographically fixable photonic crystal. *Nat. Photonics* **3**, 534-540 (2009).
8. Ohtsuka, Y., Seki, T. & Takeoka, Y. Thermally Tunable Hydrogels Displaying Angle-Independent Structural Colors. *Angew. Chem. Int. Ed.* **54**, 15368-15373 (2015).
9. Zhang, Y. et al. Super-Elastic Magnetic Structural Color Hydrogels. *Small* **15**, e1902198 (2019).
10. Li, K. et al. Facile full-color printing with a single transparent ink. *Sci. Adv.* **7**, eabh1992 (2021).
11. Patel, B. B. et al. Tunable structural color of bottlebrush block copolymers through direct-write 3D printing from solution. *Sci. Adv.* **6**, eaaz7202 (2020).

12. Boyle, B. M., French, T. A., Pearson, R. M., McCarthy, B. G. & Miyake G. M. Structural color for additive manufacturing: 3D-printed photonic crystals from block copolymers. *ACS Nano* **3**, 3052-3058 (2017).

13. Liu, Y. et al. Structural color three-dimensional printing by shrinking photonic crystals. *Nat. Commun.* **10**, 4340 (2019).

14. Liao, J. et al. 3D-printable colloidal photonic crystals. *Mater. Today* **56**, 29-41 (2022).
