# Supplementary material for: Continuous resin refilling and hydrogen bond synergistically assisted 3D structural color printing
Source: Nat Commun. 2022 Nov 19;13:7095. doi: 10.1038/s41467-022-34866-6 (PMC9675848; doi:10.1038/s41467-022-34866-6)
Supplement: Supplementary file 3 — Description of Additional Supplementary Files [file 41467_2022_34866_MOESM3_ESM.pdf]

Description of Additional Supplementary Files

File Name: Supplementary Movie 1
Description: Real-time monitoring of the continuous DLP 3D printing process of 3D Lego brick structure.

File Name: Supplementary Movie 2
Description: 3D PCs structural coloration process of printed bear structures.

File Name: Supplementary Movie 3
Description: Real-time monitoring of the continuous DLP 3D printing process of 3D pyramidlike, cylinder grid, ring, gyroid and box with twisted internal structures.
